# Extreme R-CNN: Few-Shot Object Detection via Sample Synthesis and Knowledge Distillation

**DOI:** 10.3390/s24237833

**Published:** 2024-12-07

**Authors:** Shenyong Zhang, Wenmin Wang, Zhibing Wang, Honglei Li, Ruochen Li, Shixiong Zhang

**Affiliations:** 1 School of Computer Science and Engineering, Macau University of Science and Technology, Macau 999078, China; 2009853gia30007@student.must.edu.mo (S.Z.); 2202853gia30003@student.must.edu.mo (Z.W.); 2009853gia30001@student.must.edu.mo (H.L.); lirc@bohuauhd.com (R.L.); 2009853gia30002@student.must.edu.mo (S.Z.); 2 School of Computer Technology, Beijing Institute of Technology, Zhuhai 519088, China; 3 Artificial Intelligence and Big Data College, Chongqing Polytechnic University of Electronic Technology, Chongqing 401331, China; 4 Guangdong BOHUA UHD Video Innovation Center Co., Ltd., Shenzhen 518172, China

**Keywords:** two-stage fine-tuning approach, few-shot object detection, synthesizing sample, knowledge distillation, triplet loss

## Abstract

Traditional object detectors require extensive instance-level annotations for training. Conversely, few-shot object detectors, which are generally fine-tuned using limited data from unknown classes, tend to show biases toward base categories and are susceptible to variations within these unknown samples. To mitigate these challenges, we introduce a Two-Stage Fine-Tuning Approach (TFA) named Extreme R-CNN, designed to operate effectively with extremely limited original samples through the integration of sample synthesis and knowledge distillation. Our approach involves synthesizing new training examples via instance clipping and employing various data-augmentation techniques. We enhance the Faster R-CNN architecture by decoupling the regression and classification components of the Region of Interest (RoI), allowing synthetic samples to train the classification head independently of the object-localization process. Comprehensive evaluations on the Microsoft COCO and PASCAL VOC datasets demonstrate significant improvements over baseline methods. Specifically, on the PASCAL VOC dataset, the average precision for novel categories is enhanced by up to 15 percent, while on the more complex Microsoft COCO benchmark it is enhanced by up to 6.1 percent. Remarkably, in the 1-shot scenario, the AP50 of our model exceeds that of the baseline model in the 10-shot setting within the PASCAL VOC dataset, confirming the efficacy of our proposed method.

## 1. Introduction

Few-shot object detection is a task that poses significant challenges and deserves extensive research. Object detection forms the foundation for tasks such as intelligent surveillance and unmanned driving. To achieve better performance, we usually use a large annotated training dataset, which needs more compute resources and expensive labeled costs. Labeling samples for object-detection tasks is both time-consuming and requires significant human effort. In some scenarios, it is difficult to obtain samples for object detection, such as medical images of rare diseases and photos of rare animals and plants. In certain applications that rely on sensor-collected data, amassing a large amount of image data for rare events in a short period of time can be challenging. However, few-shot object detection only needs a few samples (usually less than 30 samples) to train the model. As a result, in recent years, an increasing number of researchers have focused their efforts on few-shot object detection.

Human learning also starts with a small number of samples and is an incremental process. When children learn new knowledge, they should not forget their existing knowledge, so our approach is also an incremental learning approach. We attempt to ensure that the detector does not forget the previously learned benchmark samples when learning the few-shot samples of unknown classes.

Few-shot object-detection models are capable of recognizing novel categories using only a limited number of samples, a feature that is essential for the rapid deployment of object-detection models in dynamic environments. Two primary challenges arise in the context of few-shot object detection: (1) Insufficient samples: Training a comprehensive model from scratch is impractical due to the lack of sufficient data. To mitigate this, transfer learning is commonly employed, where a model is first pre-trained on base categories with ample data and then fine-tuned on the few-shot dataset. However, the small sample size during fine-tuning can lead to overfitting, which undermines the model’s generalization ability. (2) Performance decline on base categories: After fine-tuning, while the model gains the ability to identify novel categories, its performance in recognizing the base categories often declines.

This paper addresses the aforementioned challenges through two key strategies: (1) Enhancing sample size and diversity: In scenarios with limited samples, we aim to increase the sample size and diversity through data synthesis, thereby reducing the risk of overfitting. (2) Preserving recognition ability: We use knowledge distillation to maintain the model’s ability to recognize base categories as much as possible during the fine-tuning process.

Recently, Knowledge Distillation (KD) [1] has been introduced as a framework to compress models, enhancing the efficiency of training deep neural networks through a teacher–student learning paradigm. In this paper, we adopt a two-stage fine-tuning method to train a few-shot object detector, leveraging intermediate representations from the teacher model to guide and improve both the training process and the final performance of the student model.

Sample synthesis can reduce overfitting in few-shot object detection. For object detection, training has to extract structure from very large, highly redundant datasets using enormous amounts of computation. The amount of training samples for an object detector is a crucial factor that significantly affects the robustness and performance of the detector. We crop instances of objects from the original samples and synthesize new samples by data-augmentation methods such as adding noise to the instances. The localization capability of the detector is motivated by randomly translating the target positions in the synthetic samples. Enhancing the instances with augmentation can improve the classification performance of the detector. Overfitting of the detector can be effectively mitigated by increasing the diversity of the sample distribution through sample synthesis. The detector’s overall performance is significantly enhanced.

To enhance instance-level discriminative feature representations on synthetic samples, we integrate contrastive learning into our framework. Contrastive learning has demonstrated its effectiveness in a variety of tasks, including classification [2], identification [3], and self-supervised learning [4,5,6]. This learning paradigm is particularly adept at generating representations that can effectively differentiate between similar instances, even when labeled data are scarce, by ensuring that representations of similar data points are closely aligned while those of dissimilar points are distinctly separated. We argue that object representations refined through contrastive learning, which emphasize intra-class compactness and inter-class distinctions, can reduce the likelihood of misclassifying novel objects as belonging to similar classes. By combining sample synthesis with contrastive learning, we aim to produce a more diverse and comprehensive set of training instances, enabling the model to learn finer-grained and context-sensitive representations, thus boosting its overall performance and robustness.

**Figure 1:**
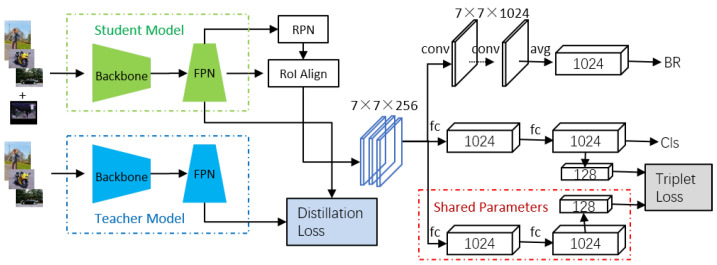
Overview of the Extreme R-CNN Framework. In the base training phase, the whole object detector, comprising the backbone, FPN, and head network, is jointly trained on the base classes. During this initial stage, the parameters of the teacher model are trained on these base classes. In the few-shot fine-tuning phase, the backbone and FPN are guided by the intermediate representations learned by the teacher model, which enhances the training process and boosts the representation capabilities of the student model. Furthermore, the box predictor is fine-tuned on a balanced dataset that includes both base and novel classes, augmented with synthesized samples. We decouple the classification and box regression of RoIs in the head network. For classification, we introduce a Siamese network and a triplet loss. Note that the RPN branch is not visualized for simplicity.

In this paper, we present Extreme R-CNN, shown in Figure 1, a model tailored for object detection in situations where the amount of training samples is extremely restricted. We begin by decoupling the regression and classification branches of the Faster R-CNN [7] head network. Initial training is conducted on base categories that are rich in data, followed by fine-tuning on a few-shot dataset comprising both base and novel categories. To boost the model’s performance in scenarios with extremely limited raw samples, we utilize data-augmentation techniques to produce new synthetic samples based on object instances. Knowledge distillation is applied to guide the training of the model’s backbone and Feature Pyramid Network (FPN). Additionally, on the classification branch of the head network, we implement Siamese networks and triplet loss to improve the model’s classification performance for Regions of Interest (RoIs). Ultimately, our model achieves AP50 scores in the 1-shot setting on the PASCAL VOC [8] dataset that exceed those of the baseline (TFA [9]) in the 10-shot setting.

To the best of our knowledge, our work is the first to integrate sample synthesis and knowledge distillation within Faster R-CNN for few-shot object detection. We refer to the model as Extreme R-CNN, which utilizes a straightforward yet effective two-stage fine-tuning approach (TFA) for few-shot object detection. Extensive experiments demonstrate the effectiveness of our straightforward design, which improves AP across all shot settings (1, 2, 3, 5, and 10 shots), achieving up to a +15% gain in average precision on the PASCAL VOC benchmark and a +6.1% gain on the challenging Microsoft COCO [10] benchmark compared to the baseline TFA.

Our contributions are threefold:

1. By synthesizing new samples through the data augmentation of object instances and fine-tuning the modified Faster R-CNN model with these samples, we significantly improve the model’s average precision for novel categories.
2. Utilizing knowledge distillation, we enhance the model’s recognition capability for novel categories while ensuring that its performance on base categories remains unaffected.
3. We validated the effectiveness of our approach in enhancing the performance of few-shot object-detection models through extensive experiments on the Microsoft COCO and PASCAL VOC datasets.

## 2. Related Work

### 2.1. Few-Shot Object Detection

Few-shot learning (FSL) aims to recognize new categories using only a limited number of labeled samples. The core concept behind FSL is to transfer knowledge from data-rich base categories to novel categories with few examples. In contrast, few-shot object detection presents a greater challenge, as it involves both classification and localization, and it remains underexplored. Few-shot object detection must identify novel objects and localize them within an image using only a few labeled samples. Two main approaches address the challenging problem of few-shot object detection (FSOD). Existing models can primarily be categorized into the following two types based on their architecture: (1) Single-branch based models [9,11,12,13,14]. These models aim to train object detectors using long-tailed training datasets that include both sample-rich base categories and sample-poor novel categories. The number of classes to be detected determines the architecture of the detector’s last classification layer. In [9], Wang et al. demonstrate that a simple two-stage fine-tuning approach (TFA) outperforms more complex meta-learning methods. To address the imbalance in the training set, two primary strategies are employed: re-sampling [9] and reweighting [15]. Subsequent studies have introduced a variety of techniques to enhance few-shot object detection (FSOD). These advancements include multi-scale positive sample refinement [12], which improves the quality of positive samples across different scales; image hallucination [13], which generates additional training data to augment the limited samples available; contrastive learning [11], which helps in learning more discriminative feature representations by contrasting positive and negative samples; and the incorporation of linguistic semantic knowledge [14], which leverages external information to improve model understanding and generalization. (2) Two-branch based models [16,17,18,19,20,21,22]. These models process support and query images concurrently through a Siamese network architecture and compute the similarity between image proposal regions and few-shot samples for detection. In [21], Kang et al. were the first to introduce a feature-reweighting module designed to aggregate features from the support and query sets. Multifeature fusion networks [16,18,22,23,24] have been introduced to achieve more robust feature aggregation. In [18], Han et al. utilized attention mechanisms to focus on foreground regions and performed feature alignment between the two inputs. In [17], Graph Convolutional Networks (GCNs) were applied to facilitate mutual adaptation between the two branches of the network. Other works [20,25,26,27] have employed more advanced nonlocal attention or transformer mechanisms [28,29,30] to enhance similarity learning between two inputs. Despite these advances, many studies still consider fine-tuning-based approaches as strong baselines that often outperform meta-learning methods. This suggests that the representation of features learned from base categories can be effectively transferred to novel categories, and simple modifications to the box predictor can lead to significant performance improvements [31]. We discourage the use of complex algorithms, as they tend to overfit and yield poor test results in few-shot object detection (FSOD). Our insight is that the degradation of average precision (AP) for novel categories primarily results from misclassifying novel instances as similar base categories. To address this issue, we leverage a Siamese network along with triplet loss to develop more discriminative object proposal representations, without increasing the model’s complexity. This approach enhances the model’s ability to distinguish between novel and base categories, thereby improving classification accuracy for novel instances.

In this paper, we utilize TFA, a form of transfer learning. In addition, we introduce knowledge-distillation and sample-synthesis techniques.

### 2.2. Sample Synthesis

Sample synthesis, also known as data augmentation or synthetic data generation, is a vital technique employed in deep learning to enhance the quality and quantity of training datasets. This approach tackles issues such as data scarcity, class imbalance, and overfitting. Sample synthesis reduces both costs and time, as collecting and labeling numerous real-world samples in any domain can be both tedious and resource intensive. In [32], Jonas Nilsson et al. enhanced their pedestrian-detection system by generating augmented images, which involved overlaying virtual pedestrians onto real image backgrounds. When these augmented data were combined with raw data, the detection accuracy was significantly improved compared to using raw data alone. In [33], Sebastiaen C. Wong et al. introduced two novel methods for data augmentation: synthetic data sampling and data warping. Synthetic data sampling generated additional samples within the feature space, while data warping created new samples within the data space. To evaluate the effectiveness of these methods, the researchers utilized convolutional backpropagation neural networks. Their findings indicated that data warping yielded better results than synthetic data sampling, provided that reasonable transformations could be identified for the data. In [34], Joseph Lemle et al. implemented smart augmentation, where they automatically combined images to enhance regularization. This method learns the optimal way to merge two or more examples from the same class, thereby identifying the most effective augmentation technique for a given dataset. In [35], Jia Shijie et al. assessed the effectiveness of different data-augmentation methods in tasks of image classification. They tested methods such as rotating, flipping, color jittering, PCA jittering, adding noise, and using Generative Adversarial Networks (GANs). Their conclusions stated that rotation, flipping, GANs, and cropping outperformed the other methods. In [36], Fujita et al. presented a novel data-augmentation strategy utilizing image transformations. By adding noise to the images, they were able to generate new samples, thereby alleviating the scarcity of data within the existing feature space. The model trained with the augmented dataset achieved better performance compared to the model trained with the raw dataset. In [37], Cheng Lei et al. evaluated the impact of various augmentation parameters on the performance of deep learning models. These parameters included the type of augmentation method, the number of samples per class in the original dataset, and the augmentation rate. Their findings revealed that geometric transformations do not consistently enhance model performance; In fact, combining two geometric transformations often resulted in diminished generalization capabilities. However, they observed that integrating geometric transformations with photometric transformations yielded significantly better outcomes. Medical image analysis often encounters the challenge of limited data, as raw images of specific diseases are not consistently plentiful. In [38], Abdulaziz Namozov et al. demonstrated that data-augmentation techniques can significantly enhance the classification capabilities of models by expanding and diversifying a computed tomography (CT) scan image dataset.

Unlike the aforementioned data-augmentation techniques, our approach involves cropping instances of novel categories from images. We then apply color channel transformations, add Gaussian noise, perform random translation, and more to generate multiple new samples, thereby increasing the variance of novel categories within the dataset.

### 2.3. Knowledge Distillation

Knowledge distillation is a technique designed to produce a smaller, more efficient model (student model) that can achieve comparable accuracy to larger models (teacher models) while being less computationally demanding and more readily deployable on resource-constrained devices or systems. This procedure aims to train a smaller model to mimic the output or behavior of larger models by distilling their knowledge, or valuable information. In [39], Bucila et al. presented an algorithm to train a single deep neural network by mimicking the output of a combination of models. In [40], Ba and Caruana compressed deep neural networks into shallower but broader architecture by applying the idea of Bucila et al. In [1], Hinton et al. used the teacher model’s output as a ‘soft label’ to train the student model and introduced temperature-scaled cross-entropy loss to replace the L2 loss. Compared to [39], they apply knowledge distillation as a more general method. In [41], Romero et al. employed a two-stage strategy to train the student model by using the output of the teacher model’s intermediate layers as ‘hints’.

Inspired by the aforementioned works, during the fine-tuning phase of training the few-shot object detector, we utilize the backbone and FPN network pre-trained on data-rich base categories from the first stage as the teacher model.

## 3. Method

### 3.1. Overview of Our Proposed Model (Extreme R-CNN)

In this work, we propose the Extreme R-CNN model, designed for few-shot object-detection scenarios based on the Faster R-CNN architecture. Our approach falls under the category of two-stage fine-tuning approaches in the field of few-shot object detection. Although existing few-shot object-detection models [9,11,12,18] have achieved promising results, they often experience a significant decrease in average precision (AP) for base categories when fine-tuning on datasets that include both base and novel categories.

In contrast to existing few-shot learning approaches, our approach focuses on a two-stage fine-tuning strategy that integrates sample synthesis and knowledge distillation. Our approach is designed to handle few-shot scenarios more effectively by augmenting the training set with synthetic data and using knowledge distillation to transfer the learned representations from the pre-trained model in the first stage. This combination helps improve generalization and robustness, especially when the number of raw samples is extremely limited.

Figure 1 illustrates an overview of our model. It is built upon the Faster R-CNN object-detection architecture. In Section 3.2, we detail how we synthesize samples for novel categories, and in Section 3.3 we explain the feature-distillation process for base categories. The architecture of the model’s head network is presented in Section 3.4 and Section 3.5.

### 3.2. Synthesizing Samples

**Figure 2:**
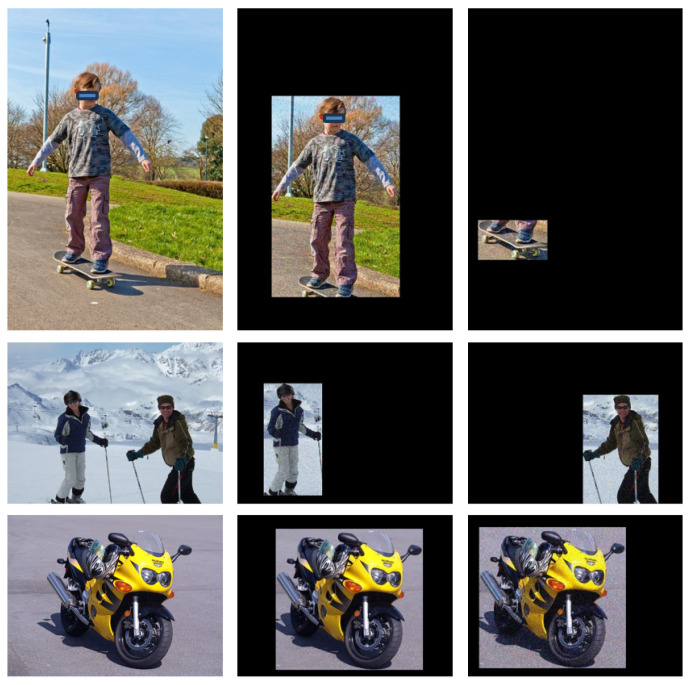
A sample graph of the synthesized samples.

In the fine-tuning phase, we synthesize samples, taking inspiration from diffusion models and generative adversarial networks. This approach ensures the generalization performance of the model even when the number of raw samples is extremely limited. We crop instances from raw images and apply data-augmentation techniques including RGB color channel transformation, adding Gaussian noise, and introducing salt-and-pepper noise. The augmented instances are then subjected to random zero-padding to generate new samples that match the size of the original image. A sample graph of the synthesized samples is illustrated in Figure 2.

From Figure 2, it can be seen that the first column shows the raw samples and the second column shows the instances cut from these raw samples. These instances are then augmented with color channel transformations, Gaussian noise, and salt and pepper noise to generate the synthesized samples shown in the second column. The locations of these instances are randomly shifted with respect to the raw samples. Similarly, the third column shows either different instances or the same instance synthesized using various data-augmentation methods. Both the data-augmentation technique and the position translation are applied randomly during each sampling.

Compared to commonly used data-augmentation methods, our sample-synthesis method only enhances the instances, increasing the number and diversity of training samples, and improves the generalization performance of the model during fine-tuning. At the same time, by randomly shifting the locations of the instances, this helps to enhance the class-agnostic object-localization capability of the model.

### 3.3. Knowledge Distillation for Base Categories

Knowledge Distillation (KD) is a machine learning technique that transfers the knowledge from a complex model, a.k.a. the teacher model, to a simpler model, referred to as the student model. When fine-tuning on novel and base categories in a few-shot setting, we use a backbone and Feature Pyramid Network (FPN) as the teacher model. We employ a hint loss to encourage the student model to mimic the feature representations learned by the intermediate layers of the teacher model. The weights obtained from the initial training phase are used in the teacher model to guide the subsequent fine-tuning phase. This process helps ensure that the performance on the base category remains robust. The mathematical formulation of the hint loss is provided in Equations (1) and (2).

(1) $$f\left(x_{i}\right)=\left\{\begin{array}{cc} 1 & \mathrm{if}\;x_{i}\in D_{Base\;Categories}\\ 0 & \mathrm{if}\;x_{i}\notin D_{Base\;Categories} \end{array}\right.\;$$

(2) $$\;L_{\;\mathrm{Hints}}=\frac{1}{\sum_{i=1}^{N}f\left(x_{i}\right)}\sum_{i=1}^{N}f\left(x_{i}\right)\times {∥F_{T}\left(x_{i}\right)-F_{S}\left(x_{i}\right)∥}^{2}$$

### 3.4. Decoupled Classification and Regression Heads

Decoupling the classification and regression head networks significantly enhances the overall accuracy of object-detection models [42,43], particularly in few-shot object-detection scenarios. This architectural choice allows each task—classification and regression—to be optimized independently, leading to more specialized and effective sub-networks for their respective objectives. In R-CNN-based detectors for regression and classification tasks, there are two commonly used head architectures: the convolutional head (conv-head) and the fully connected head (fc-head). Generally, the conv-head is better suited for localization tasks, while the fc-head excels in classification tasks. While the fc-head has greater spatial sensitivity than conv-head, which aids in distinguishing between complete and partial objects, it may not be as robust as the conv-head when it comes to the effective regression of the whole object. Overall, fully connected heads are ideal for classification tasks, while convolutional heads excel at regression. During the fine-tuning phase, which relies on a finite sample set, decoupling the classification and regression heads ensures that class-agnostic bounding box regression is largely unaffected by small sample sizes. In order to enhance the classification performance during fine-tuning in the presence of insufficient samples, Siamese network and triplet loss are introduced to allow the classification head to learn a feature space that is more easily classifiable, thus improving the classification accuracy. As a result, the decoupled head architecture is better suited for few-shot object-detection scenarios.

### 3.5. Using Siamese Network and Triplet Loss in Classification Head

Deep learning models tend to overfit when there are insufficient sample data. After decoupling the classification and regression head networks, we introduce the Siamese network and triplet loss during fine-tuning with the few-shot dataset. This approach minimizes the distance between positive examples (samples from the same class) and maximizes the distance between negative examples (samples from different classes), thus enabling the model to learn a more discriminative feature space. The architecture of the Siamese network is shown in Figure 1, and the formula for the triplet loss function is provided as Equation (3).

(3) $$L_{\mathrm{Triplet}}=\max\left(\alpha +\frac{f(A)\cdot f(N)}{∥f(A)∥∥f(N)∥}-\frac{f(A)\cdot f(P)}{∥f(A)∥∥f(P)∥},0\right)$$

where α is a positive constant known as the margin.

To summarize, the overall loss of the Extreme R-CNN model consists of the loss from the Faster R-CNN model, the distillation loss from the backbone and FPN, and the triplet loss from the Siamese network. The total training loss of the detector is denoted as *L*_Total_ in Equation (4)

(4) $$L_{\mathrm{Total}}=\lambda_{d}L_{\;\mathrm{Hints}}+L_{\mathrm{Faster}\;R-\mathrm{CNN}}+\lambda_{t}L_{\mathrm{Triplet}}$$

here, $\lambda_{d}$ and $\lambda_{t}$ are hyperparameters that indicate the weights of $L_{\;\mathrm{Hints}}$ and $L_{\;\mathrm{Triplet}}$, respectively.

## 4. Experiments

We have conducted extensive experiments on both PASCAL VOC and Microsoft COCO benchmarks. Extreme R-CNN demonstrates significant performance improvements over all fine-tuning-based approaches, with a substantial margin in any shot scenario across all dataset splits. We strictly adhere to a consistent few-shot detection dataset-construction and -evaluation protocol [9,12,21,23] to ensure a fair and direct comparison. In this section, we begin by detailing the few-shot detection setup employed in our study. We then present a comprehensive comparison of our approach with contemporary few-shot detection methods on the PASCAL VOC and Microsoft COCO benchmarks. Finally, we conduct ablation experiments to analyze the impact of various components and design choices on the performance of our model.

### 4.1. Experiment Configuration

#### 4.1.1. Datasets

Our method has been evaluated on the benchmark datasets PASCAL VOC and Microsoft COCO, following the protocols established by previous works [9,21]. To ensure a fair comparison, we adopt the data splits and annotations as described by TFA [9]. For the PASCAL VOC dataset, the 20 categories are divided into three distinct groups, each consisting of 15 base and 5 novel categories. The training is conducted using all available data from the base categories in the PASCAL VOC 2007 and 2012 trainval sets. In the K-shot settings, where K takes values of 1, 2, 3, 5, and 10, we randomly sample instances from previously unseen novel categories during training. In line with prior studies [9,21,23], we use the same three random partitions of base and novel categories, referred to as Novel Split 1, 2, and 3, as outlined in [21], to maintain consistency across experiments. Regarding the Microsoft COCO dataset, we define 60 categories that do not overlap with PASCAL VOC as our base categories, while the remaining 20 categories are designated as novel categories. We evaluate the detection performance for 10-shot and 30-shot settings on 5 K images from the Microsoft COCO 2014 validation dataset, ensuring a comprehensive evaluation of the few-shot learning capabilities of our model.

#### 4.1.2. Evaluation Metrics

For the PASCAL VOC dataset, we present the Average Precision (AP) at an Intersection over Union (IoU) threshold of 0.5 for both base categories (denoted as bAP) and novel categories (denoted as nAP), evaluated on the PASCAL VOC 2007 test set. For the Microsoft COCO dataset, we provide the mean AP averaged across IoU thresholds ranging from 0.5 to 0.95 for novel categories (nAP), along with the AP at an IoU threshold of 0.75 for the novel categories (nAP75); these metrics are evaluated on a subset of 5K images from the Microsoft COCO 2014 validation dataset.

#### 4.1.3. Implementation Details

Our model’s code is built using the Detectron2 [44] framework, employing a ResNet-101 [45] backbone along with a Feature Pyramid Network (FPN) [46]. All experiments are conducted in JupyterLab with an NVIDIA Tesla V100-SXM3 GPU (NVIDIA, Santa Clara, California, USA. NVIDIA Operations in Macau) We train all models using standard SGD with a momentum of 0.9 and a weight decay of 1 × 10^−4^. The learning rate is set to 0.02 for training base categories, and 0.001 for training novel categories. The batch size is 16 for all training runs. The model was fine-tuned for {3000, 5000, 6000, 10,000, 12,000} iterations for K = {1, 2, 3, 5, 10} shots in the PASCAL VOC dataset, and for {30,000, 40,000} iterations for K = {10, 30} shots in the Microsoft COCO dataset. Unless otherwise specified, we maintain the same hyperparameters as those used in Faster R-CNN.

### 4.2. Main Results

#### 4.2.1. PASCAL VOC

As shown in Table 1, the Extreme R-CNN model achieves the second best average AP among the existing methods, trailing only the VFA model. Extreme R-CNN achieves the best results in 4 out of 15 settings and the second best in 11 out of 15 settings. In Novel Set 1, Extreme R-CNN outperforms the baseline (TFA w/cos) by a margin ranging from 15.8% to 72.5%. Notably, our 1-shot result even surpasses the baseline’s (TFA w/cos) 10-shot results (57% vs. 56%), indicating that Extreme R-CNN is particularly effective in data-scarce scenarios. Moreover, our improvement is consistently stable across all Novel Split sets, demonstrating that Extreme R-CNN is unbiased towards any specific subset of categories and has strong generalization capabilities. Additionally, Extreme R-CNN achieves a mean average precision of 54.9%, improving upon the baseline (TFA w/cos) by 15 percentage points (from 39.9% to 54.9%), which further underscores its effectiveness. Meanwhile, as shown in Figure 1, our model performs slightly worse than the VFA method. We note that VFA is a meta-learning paradigm that employs variational feature aggregation on samples. As suggested, this method of augmenting samples through variational feature aggregation may be more suitable for meta-learning. However, VFA requires training multiple models using a variational feature-aggregation dataset and then selecting the optimal model, making its training process more complex and computationally demanding compared to our approach.

#### 4.2.2. Microsoft COCO

The few-shot detection results for Microsoft COCO are presented in Table 2. Our Extreme R-CNN achieves the second best nAP among the fine-tuning-based methods under the same testing protocol and metrics. Compared to the baseline (TFA w/cos), our model shows improvements of +6.5% nAP in the 10-shot scenario and +5.6% nAP in the 30-shot scenario. Based on the results presented in Table 1 and Table 2, our Extreme R-CNN model achieves the second best average AP on the PASCAL VOC dataset and also achieves the second best results on the Microsoft COCO dataset. This consistent performance across the two datasets highlights the effectiveness of our model in enhancing the performance of few-shot object detection. The improvement in the 10-shot setting is more pronounced than in the 30-shot setting, suggesting that our proposed model provides a more significant benefit when the sample size is smaller. Similarly, in Figure 1, our method performs better than the DeFRCN method, while in Figure 2, our method performs slightly worse. Compared to the Microsoft COCO dataset, PASCAL VOC has significantly fewer categories and sample sizes. This suggests that the effectiveness of our method in improving model performance decreases as the number of categories and sample size increases. This observation highlights the need for further research to enhance our method’s performance in more complex datasets.

### 4.3. Ablation Study

To decouple the classification and regression processes within the detector’s head network during the fine-tuning stage, we modified the Faster R-CNN model by bifurcating the head network into two distinct branches: one specialized for classification and the other for regression. Subsequently, we performed base training on this modified Faster R-CNN model using the base categories dataset, and then proceeded with fine-tuning on the novel categories dataset. The results from the fine-tuning process are depicted in the third row of Table 3.

We perform ablation experiments to demonstrate the effectiveness of primary components proposed in Extreme R-CNN. Our work proposed three components, synthesizing sample, knowledge distillation, Siamese network, and triplet loss. We utilize the ResNet-101 with a Feature Pyramid Network (FPN) as backbone, and gradually incorporate each component into the baseline model according to the parameter configurations detailed in Section 4.1. Unless otherwise specified, all ablation experiments are performed using PASCAL VOC Novel Split 1 and the results are shown in Table 3.

#### 4.3.1. Synthesizing Samples

To verify the effectiveness of synthesizing samples, we utilized synthesized data for fine-tuning the baseline TFA model. As demonstrated in the fourth row of Table 3, this approach improved the average score of nAP50 by 8.1% (from 51.1% to 59.2%). This indicates that the method of synthesizing samples is effective in enhancing the performance of an object detector trained with few-shot samples. Notably, the enhancement is more pronounced in the 1-shot scenario. We attribute this to the increased diversity brought about by data augmentation applied to the synthesized samples, which boosts the model’s generalization capability.

#### 4.3.2. Knowledge Distillation

To evaluate the impact of knowledge distillation on detector performance, we integrated a teacher model into the baseline during the fine-tuning phase. The fifth row of Table 3 presents the results of the baseline with knowledge distillation. We can observe that knowledge distillation improves the average score of nAP50 by 0.6% (from 59.4% to 60.0%). The results show that while knowledge distillation does not significantly improve the nAP50 of the model, it effectively preserves the bAP50 for the base categories without causing a significant drop. Since, in the fine-tuning phase, knowledge distillation is applied only to base categories, novel categories are unknown to the pre-trained model from the first phase. This means that the teacher model cannot guide the training of novel categories during the fine-tuning phase. Therefore, knowledge distillation does not significantly improve the recognition accuracy of the model for novel categories. The detailed data are presented in Table 4.

#### 4.3.3. Siamese Network and Triplet Loss (SNTL)

To understand the significance of SNTL, we incorporate the Siamese network into the classification head network of Faster R-CNN during the fine-tuning stage. Triplet loss is used to ensure that the distance between RoIs of the same class is closer than the distance between RoIs of different classes by satisfying a predefined margin. This enhances the representation space of the classification head network for RoIs, thus improving the overall classification performance. As shown in the sixth row of Table 3, the implementation of the SNTL improves the average nAP50 score by 2.8% (from 60.0% to 62.8%).

## 5. Conclusions and Future Work

In this paper, we propose Extreme R-CNN, an extension of Faster R-CNN specifically designed for few-shot object detection. Extreme R-CNN adopts a two-stage fine-tuning approach (TFA) that integrates knowledge distillation and the Siamese network. During the fine-tuning phase, the model is trained using synthetic samples. The effectiveness of our method in detecting objects with few shots has been validated through a series of extensive experiments performed on the PASCAL VOC and Microsoft COCO datasets. In particular, Extreme R-CNN is able to quickly learn to perform object detection and recognize new classes even in extremely limited data samples, such as in a one-shot scenario. Therefore, our model has important applications in situations where it is difficult to obtain multiple samples.

There are two major limitations of our approach that could be addressed in future studies. First, as the number of samples in each category increases, the effect of sample synthesis on improving model performance diminishes. In other words, with a large sample size, the impact of sample synthesis on enhancing model performance is not significant. Second, synthesizing new samples by cutting and pasting instances from sample images is not effective for identifying small objects within the samples. In the future, we will continue to investigate the impact of sample synthesis on improving model performance from multiple perspectives, including different synthesis methods, and the relationship between the number of synthesized samples and model performance. Additionally, we will explore how to ensure that the model’s ability to recognize base categories does not decrease significantly when new categories are added. We also plan to apply our method to different models and datasets.

## Figures and Tables

**Table 1:** Experimental results on the VOC dataset. We evaluate the performance of Extreme R-CNN (AP50) on three different splits. The best results are in red and the second best results are in blue.

Method/Shot	Novel Split 1					Novel Split 2					Novel Split 3					Avg.
	**1**	**2**	**3**	**5**	**10**	**1**	**2**	**3**	**5**	**10**	**1**	**2**	**3**	**5**	**10**	
TFA w/cos (Baseline)	39.8	36.1	44.7	55.7	56	23.5	26.9	34.1	35.1	39.1	30.8	34.8	42.8	49.5	49.8	39.9
FSCE	44.2	43.8	51.4	61.9	63.4	27.3	29.5	43.5	44.2	50.2	37.2	41.9	47.5	54.6	58.5	46.6
DeFRCN	53.6	57.5	61.5	64.1	60.8	30.1	38.1	47.0	53.3	47.9	48.4	50.9	52.3	54.9	57.4	51.9
VFA	57.7	64.6	64.7	67.2	67.4	41.4	46.2	51.1	51.8	51.6	48.9	54.8	56.6	59.0	58.9	56.1
Extreme R-CNN (Ours)	57.0	58.3	62.7	65.3	66.2	40.2	46.4	47.8	52.4	53.8	49.5	52.3	54.4	57.3	59.4	54.9
Improvement (%)	+43.2	+61.5	+40.3	+17.2	+18.2	+71.1	+72.5	+40.2	+49.3	+37.6	+60.1	+50.3	+27.1	+15.8	+19.3	+37.5

**Table 2:** Experimental results on the Microsoft COCO dataset. The backbone is the same as in Table 1. The best results are in red and the second best results are in blue.

Method	nAP 10	nAP 30
LSTD [47]	3.2	6.7
FSRW	5.6	9.1
MetaDet	7.1	11.3
Meta-RCNN	8.7	12.4
MPSR	9.8	14.1
TFA w/cos (Baseline)	10.0	13.7
FSCE	11.9	16.4
FADI	12.2	16.1
VFA	16.2	18.9
DeFRCN	18.5	22.6
Extreme R-CNN (ours)	16.5	19.3

**Table 3:** Ablation for primary components proposed in Extreme R-CNN.

Method	SS	KD	SNTL	Novel AP50 Split 1			
				**1**	**5**	**10**	**Avg.**
TFA w/cos (Baseline)	-	-	-	39.8	55.7	56.0	50.5
TFA w/cos (Our reimpl.)	✕	✕	✕	40.2	55.9	57.2	51.1
Extreme R-CNN (Ours)	√	✕	✕	51.1	62.7	64.3	59.4
	√	√	✕	51.8	63.3	65.0	60.0
	√	√	√	57.0	65.3	66.2	62.8

**Table 4:** Base category forgetting comparison on PASCAL VOC Split 1. Prior to fine-tuning, the base AP50 achieved during base training is 81.2. Bold indicates the best value.

Method	Base AP50				Novel AP50			
	**1**	**3**	**5**	**10**	**1**	**3**	**5**	**10**
MPSR [12]	59.4	67.8	68.4	-	41.7	51.4	55.2	61.8
FSCE [11]	78.9	74.1	76.6	-	44.2	51.4	61.9	63.4
TFA w/cos [9] (Baseline)	**79.1**	77.3	77.0	-	39.8	44.6	55.7	56.0
TFA w/cos [9] (Our reimpl.)	78.8	**77.5**	**77.2**	**77.0**	40.2	45.3	55.9	57.2
TFA w/cos [9] + SS	77.5	76.7	76	75.4	51.1	56.3	62.7	64.3
TFA w/cos [9] + SS + KD	78.4	77.3	77	76.5	51.8	56.9	63.3	65.0
Extreme R-CNN (Ours)	78.2	77	76.7	76.3	**57**	**62.7**	**65.3**	**66.2**

## Data Availability

Some or all data, models or code generated or used during the study are available from the first author and the corresponding author by request.
